# Which Motor Competence Components Drive Early Motor Learning? A Phase-Based Analysis in Preschool Children

**DOI:** 10.3390/jfmk11030303

**Published:** 2026-07-30

**Authors:** Kristian Plazibat

**Affiliations:** 1 Natural Sciences School Vladimir Prelog, 10000 Zagreb, Croatia; kristian.plazibat@gmail.com; 2 Faculty of Educational Sciences, University of Slavonski Brod, 35000 Slavonski Brod, Croatia; 3 Faculty of Kinesiology, University of Zagreb, 10000 Zagreb, Croatia

**Keywords:** motor competence, motor learning, preschool children, BOT-2, phase-based learning, kinesiology program, early childhood, motor development

## Abstract

**Background**: Motor competence is an important basis for early childhood motor development and for the acquisition of new motor skills. Previous work from this research perspective showed that structured kindergarten kinesiology programs and overall BOT-2 motor proficiency are associated with phase-based motor learning outcomes. However, it remains unclear whether individual motor competence components are related differently to distinct phases of early motor learning. The aim of this study was to examine associations between selected BOT-2 motor competence components and phase-based motor learning outcomes in preschool children. **Methods**: A total of 161 preschool children participated in the study. Selected BOT-2 components were used to describe baseline motor competence. Motor learning was assessed using a standardized 7-week protocol involving previously unfamiliar kinesiology tasks and was operationalized through time to initial acquisition (F1), time to early refinement (F2), and final performance quality (K). Correlation analyses and hierarchical regression models were used, controlling for age, sex, and previous participation experience. **Results**: Motor competence components were positively interrelated and were significantly associated with learning outcomes. Higher baseline motor competence was generally associated with shorter F1 and F2 times and higher K scores. Participation experience showed the largest and most consistent associations across outcomes. Fine motor precision was independently associated with F1 and F2, while running speed and agility was retained as a predictor of F2. **Conclusions**: The findings indicate that previous structured movement experience and selected motor competence components are associated with phase-based motor learning outcomes in preschool children. These results should be interpreted as associative rather than causal and support the use of assessment-informed approaches in early childhood kinesiology programs.

## 1. Introduction

Motor development in early childhood represents an important foundation for physical activity participation, motor learning, and broader developmental outcomes [1,2,3]. During the preschool period, children progressively develop locomotor skills, object-control skills, balance, coordination, postural control, and strength, which together form the basis for more complex movement patterns and successful engagement in structured physical activity [1,4,5]. Within this developmental context, motor competence should not be viewed only as an outcome of physical activity, but also as a functional basis for acquiring and refining new motor skills [6,7,8].

Structured physical activity and organized kindergarten kinesiology programs can provide children with repeated opportunities to practice diverse movement patterns under planned and supervised conditions [3,9,10]. Previous work from this research line has shown that participation in a multilateral kindergarten exercise program can support the development of motor skills in preschool children and may be relevant for implementation within the kindergarten curriculum [11]. This earlier work provided the programmatic foundation for examining how structured movement experience is related to motor competence and later motor learning outcomes [11,12].

More recently, a phase-based approach was used to examine the acquisition of previously unfamiliar motor tasks in preschool children with different levels of previous participation experience in an organized kinesiology program [12]. In that study, motor learning was operationalized through time to Phase 1 (F1), reflecting the first successful execution of a task; time to Phase 2 (F2), reflecting initial refinement of performance; and final performance quality (K) [12]. The findings showed that children with greater previous participation experience generally reached early learning phases more rapidly and achieved higher final performance quality [12]. Baseline motor proficiency, assessed using the Bruininks–Oseretsky Test of Motor Proficiency, Second Edition (BOT-2), was also associated with shorter learning times and better final performance quality [12,13].

Although these findings provide important evidence that previous participation experience and overall motor proficiency are associated with phase-based motor learning, they do not fully explain which specific components of motor competence are most relevant for different phases of learning [12]. Motor competence is a multidimensional construct that includes fine motor precision, fine motor integration, manual dexterity, upper-limb coordination, balance, bilateral coordination, running speed and agility, and strength [8,13,14]. These components may be interrelated, but they are not necessarily functionally equivalent during the acquisition and refinement of new motor tasks [8,15].

This distinction is important from both theoretical and practical perspectives. From a motor learning perspective, initial acquisition may depend strongly on the child’s ability to organize basic movement structure, control movement direction, and perform task-relevant actions with sufficient precision [16,17,18]. Early refinement, in contrast, may require greater movement efficiency, stability, coordination, and the ability to repeat the emerging movement pattern with improved control [15,16,17]. Final performance quality may reflect an even broader integration of multiple motor competence components rather than the isolated influence of a single domain [8,15].

From a pedagogical perspective, identifying the specific motor competence components that contribute to early learning phases may support more differentiated and assessment-informed planning in early childhood kinesiology programs [12,19,20]. In many preschool settings, instructional decisions are based primarily on chronological age or general observations of motor readiness, although children of similar age may differ considerably in their motor competence profiles and previous structured movement experience [11,12,19]. Understanding which motor competence components are most closely related to F1, F2, and final performance quality may therefore help practitioners better align task selection, progression, and instructional support with children’s actual motor readiness [12,19,20].

A practical challenge in early childhood kinesiology programs is that repeated participation in structured exercise does not necessarily lead to proportional improvements in motor competence if program content is not progressively adapted to children’s developing motor readiness. Previous findings from this research line showed that a one-year multilateral exercise program had a positive effect on preschool children’s motor skills, whereas additional years of participation were associated mainly with maintenance of the acquired level rather than further proportional improvement [11]. This raises an important curriculum-related question: how can practitioners determine when children should continue consolidating basic movement elements and when they are ready to progress toward more complex motor tasks?

Addressing this question requires a more detailed understanding of the internal structure of motor competence and its relationship with early motor learning. If specific motor competence components are differentially associated with initial acquisition, early refinement, and final performance quality, then assessment of these components may help guide the timing and progression of instructional content. In this sense, identifying which motor competence components are most closely related to particular learning phases may provide practical information for designing developmentally progressive curricula, rather than relying only on age-based progression or repeated exposure to similar movement tasks.

The present study builds on two previous publications from the same research line. The first examined the effects of different multi-year physical exercise programs on motor skills in preschool children [11], while the second examined phase-based motor skill acquisition in relation to previous participation experience and baseline BOT-2 total proficiency [12]. The present study addresses a distinct question by moving beyond total motor proficiency and examining whether individual BOT-2 components show different associations with initial acquisition, early refinement, and final performance quality. This component-level approach is the main novel contribution of the manuscript. Evidence from youth sport also supports the view that specific motor experiences may be linked to selected motor competence components. For example, Amato et al. [21] reported better manual dexterity performance in young basketball players than in athletic and non-athletic peers of the same age, suggesting that specific movement experiences may be associated with particular motor competence domains.

Therefore, the aim of this study was to examine how selected baseline motor competence components are interrelated and how they are associated with different phase-based motor learning outcomes in preschool children. Specifically, the study examined associations among BOT-2-derived motor competence components and their relationships with time to initial acquisition (F1), time to early refinement (F2), and final performance quality (K). We hypothesized that motor competence components would be positively interrelated and significantly associated with phase-based learning outcomes. We further expected that, after controlling for age, sex, and previous participation experience, specific motor competence components would show different patterns of association with different phases of motor learning.

## 2. Materials and Methods

### 2.1. Participants

A total of 161 preschool children participated in this study. The sample consisted of 74 boys and 87 girls from the final years of kindergarten, with a mean age of 6.1 ± 0.6 years. All participants were recruited from kindergarten settings and were enrolled in regular preschool education. Children were healthy and free from diagnosed motor or developmental disorders. Participation in the study was voluntary, and written informed consent was obtained from parents or legal guardians before data collection. The study was conducted in accordance with the Declaration of Helsinki and was approved by the Ethics Committee of the Faculty of Kinesiology, University of Zagreb (Ref. No. 32/2021; approval date: 26 October 2021).

### 2.2. Research Context and Study Design

The present study is part of a broader research line examining motor competence, structured movement experience, and phase-based motor learning in preschool children. Previous work from this research line examined the effects of different multi-year physical exercise programs on motor skills in preschool children and described the organized kindergarten kinesiology program used as the background structured movement context. A later study introduced a phase-based motor learning assessment protocol and examined whether previous participation experience and baseline BOT-2 motor proficiency were associated with the speed and quality of acquiring previously unfamiliar motor tasks.

This study extends previous work from the same research line. The organized kindergarten kinesiology program and group differences in BOT-2 outcomes were described previously [11], and the phase-based motor learning protocol was introduced in a later study focused on total BOT-2 proficiency and participation experience [12]. The present manuscript retains the same participant context and phase-based protocol, but introduces a new component-level analysis of BOT-2 domains and their phase-specific associations with F1, F2, and K.

The present study used an observational analytical design. Baseline motor competence components were assessed first, and all children subsequently completed the same standardized phase-based motor learning protocol. The design was analytical in the sense that the study examined associations among already existing differences in baseline motor competence, accumulated participation experience, and standardized motor learning outcomes. No random allocation or experimental manipulation of previous experience was applied in the present analysis.

### 2.3. Previous Participation in the Organized Kindergarten Kinesiology Program

Before the standardized motor learning assessment, children differed in previous participation experience in an organized kindergarten kinesiology program. This program was implemented as an additional structured physical activity program within the kindergarten setting and was designed to support multilateral psychophysical development and the acquisition of diverse fundamental movement experiences.

One pedagogical year consisted of approximately 10 months, or 40 weeks, of organized exercise. The program was conducted three times per week, with each session lasting 60 min. The program content included basic gymnastics exercises, various forms of jumping and running, obstacle courses, manipulation of different props, and introductory elements of team sports such as football, handball, basketball, and volleyball. Through this structure, children were repeatedly exposed to locomotor, manipulative, balance, coordination, postural-control, and object-control activities.

According to institutional attendance records, children were classified by accumulated exposure to the organized kindergarten kinesiology program as having no previous participation, approximately 120 h, approximately 350 h, or approximately 470 h of structured movement exposure before the standardized motor learning assessment. This variable represented previous participation experience and was entered as an independent predictor in the hierarchical regression analyses.

### 2.4. Baseline Motor Competence Assessment

Baseline motor competence was assessed using selected subtests from the Bruininks–Oseretsky Test of Motor Proficiency, Second Edition (BOT-2). The BOT-2 is a standardized instrument for assessing motor proficiency in children and includes tasks representing both fine and gross motor domains.

For the purposes of the present analysis, selected BOT-2 subtests were grouped into the following motor competence components: Fine Motor Precision (FMP), Fine Motor Integration (FMI), Manual Dexterity (MD), Upper-Limb Coordination (ULC), Balance (BAL), Bilateral Coordination (BC), Running Speed and Agility (RSA), and Strength (STR). These components were selected because they represent distinct but potentially interrelated dimensions of motor competence that may contribute differently to the acquisition and refinement of new motor tasks.

Each component was calculated from the corresponding BOT-2 subtests and standardized as a z-score before correlation and regression analyses to enable direct comparison across motor competence domains. Detailed descriptions of the BOT-2 subtests used to define each motor competence component are provided in Supplementary Table S1.

### 2.5. Phase-Based Motor Learning Protocol and Outcome Assessment

After baseline motor competence assessment, all children completed the same standardized 7-week motor learning protocol. The protocol consisted of 14 sessions delivered twice per week and included nine previously unfamiliar kinesiology tasks. These tasks had not been practiced within the regular preschool program or within the organized kindergarten kinesiology program. The purpose of the protocol was to observe the acquisition of new motor tasks under standardized instructional and environmental conditions. The detailed session-by-session structure of the standardized 7-week motor learning protocol is provided in Supplementary Table S2 and is based on the protocol previously described in this research line [12].

All participants were exposed to the same task content, instructional procedures, equipment, practice environment, and progression structure. Task performance was video recorded during the protocol to allow delayed expert assessment of learning progression. The recordings were not sampled selectively from the protocol; instead, video material was collected systematically during the standardized learning sessions for each child and each of the nine motor tasks. Thus, for each participant, nine task-specific video recordings were available for phase-based assessment, corresponding to the nine unfamiliar motor tasks included in the protocol.

Each video recording covered the child’s continuous task performance during the allotted practice period for that task. Recording duration was determined by task complexity: approximately 2 min for simple tasks, approximately 3 min for the complex task, and approximately 4 min for the more complex task. This procedure ensured that the raters had sufficient video material to identify the first observable attainment of the predefined learning phases while keeping the assessment standardized across participants and tasks.

### 2.6. Video-Based Assessment and Rater Reliability

All phase-based motor learning outcomes were assessed through delayed expert video analysis. Video recordings were reviewed by three independent kinesiologists, each with more than five years of professional experience in preschool physical activity and motor learning. The raters evaluated each participant and each task separately using predefined operational criteria for F1, F2, and K.

For each participant, the raters reviewed the task-specific recordings from all nine motor tasks. The assessment was therefore based on nine video recordings per child, with each recording corresponding to one unfamiliar motor task from the standardized protocol. The analysis focused on identifying the first time point at which the child met the criterion for Phase 1 and the first time point at which the child met the criterion for Phase 2. Both time points were expressed in seconds of effective practice time within the recorded session, rather than total lesson duration. These values were used as F1 and F2, respectively. Final performance quality was assessed from the final recorded execution segment for each task.

Phase 1 was coded when the child demonstrated the first successful execution of the essential task structure without physical assistance. Phase 2 was coded when the child demonstrated an initially refined and repeatable execution pattern, including recognizable coordination of the main movement components, improved stability, and task-specific control. Final performance quality was rated on a 5-point scale according to recognizability of the movement pattern, coordination of essential movement segments, stability, fluency, and overall execution quality.

The task-specific criteria used to identify F1 and F2 and the general 5-point criteria used to rate final performance quality are provided in Supplementary Table S3.

Assessments were conducted independently. Disagreements in phase identification or final performance scoring were resolved through expert discussion and consensus. Inter-rater reliability was assessed using two-way random-effects intraclass correlation coefficients. Reliability was very high for both phase indicators: F1: ICC[2,1] = 0.984, ICC[2,k] = 0.995; F2: ICC[2,1] = 0.976, ICC[2,k] = 0.992. These values supported the consistency of the video-based evaluation procedure.

### 2.7. Statistical Analysis

The motor competence components were expressed as standardized composite scores. Pearson correlation coefficients were used to examine interrelationships among motor competence components and associations between motor competence components and phase-based motor learning outcomes. Correlation analyses were presented descriptively to show the bivariate structure of the data; mechanistic interpretation was reserved for the Section 4.

Hierarchical regression analyses were conducted separately for F1, F2, and K. In Step 1, age and sex were entered as control variables. In Step 2, previous participation experience was entered as an independent predictor representing accumulated structured movement exposure before the standardized motor learning assessment. In Step 3, selected BOT-2 motor competence components were entered to examine whether individual components explained additional variance in phase-based motor learning outcomes beyond age, sex, and previous participation experience.

Standardized regression coefficients (β), *p*-values, R^2^, adjusted R^2^, and ΔR^2^ values were reported. The level of statistical significance was set at *p* < 0.05. The primary analytical interest was whether specific motor competence components showed selective associations with different motor learning outcomes after accounting for age, sex, and previous participation experience.

## 3. Results

### 3.1. Interrelationships Among Motor Competence Components

Correlation analysis showed positive associations among baseline motor competence components. The strongest interrelationships were observed between fine motor precision and balance (r = 0.54, *p* < 0.001), running speed and agility and strength (r = 0.52, *p* < 0.001), and fine motor precision and strength (r = 0.51, *p* < 0.001). Additional moderate associations were identified between bilateral coordination and strength (r = 0.49, *p* < 0.001), bilateral coordination and running speed and agility (r = 0.45, *p* < 0.001), and upper-limb coordination and balance (r = 0.45, *p* < 0.001).

The correlation coefficients ranged from low to moderate, indicating that the motor competence components were related but not redundant.

### 3.2. Associations Between Motor Competence Components and Motor Learning Outcomes

The correlations between motor competence components and phase-based motor learning outcomes are presented in Table 1. Higher baseline motor competence was consistently associated with shorter learning times for F1 and F2 and with higher final performance quality.

For Phase 1 (F1), the strongest negative associations were observed for strength (r = −0.62, *p* < 0.001), fine motor precision (r = −0.59, *p* < 0.001), balance (r = −0.44, *p* < 0.001), and manual dexterity (r = −0.43, *p* < 0.001). Thus, higher scores in these components were associated with shorter time to the first successful execution.

For Phase 2 (F2), the overall magnitude of correlations was lower, but several associations remained statistically significant. The strongest associations were observed for fine motor precision (r = −0.34, *p* < 0.001), strength (r = −0.32, *p* < 0.001), and running speed and agility (r = −0.24, *p* = 0.002).

For final performance quality (K), positive associations were observed for strength (r = 0.57, *p* < 0.001), fine motor precision (r = 0.54, *p* < 0.001), manual dexterity (r = 0.42, *p* < 0.001), and balance (r = 0.42, *p* < 0.001), with additional significant associations for bilateral coordination, running speed and agility, upper-limb coordination, and fine motor integration.

**Figure 1:**
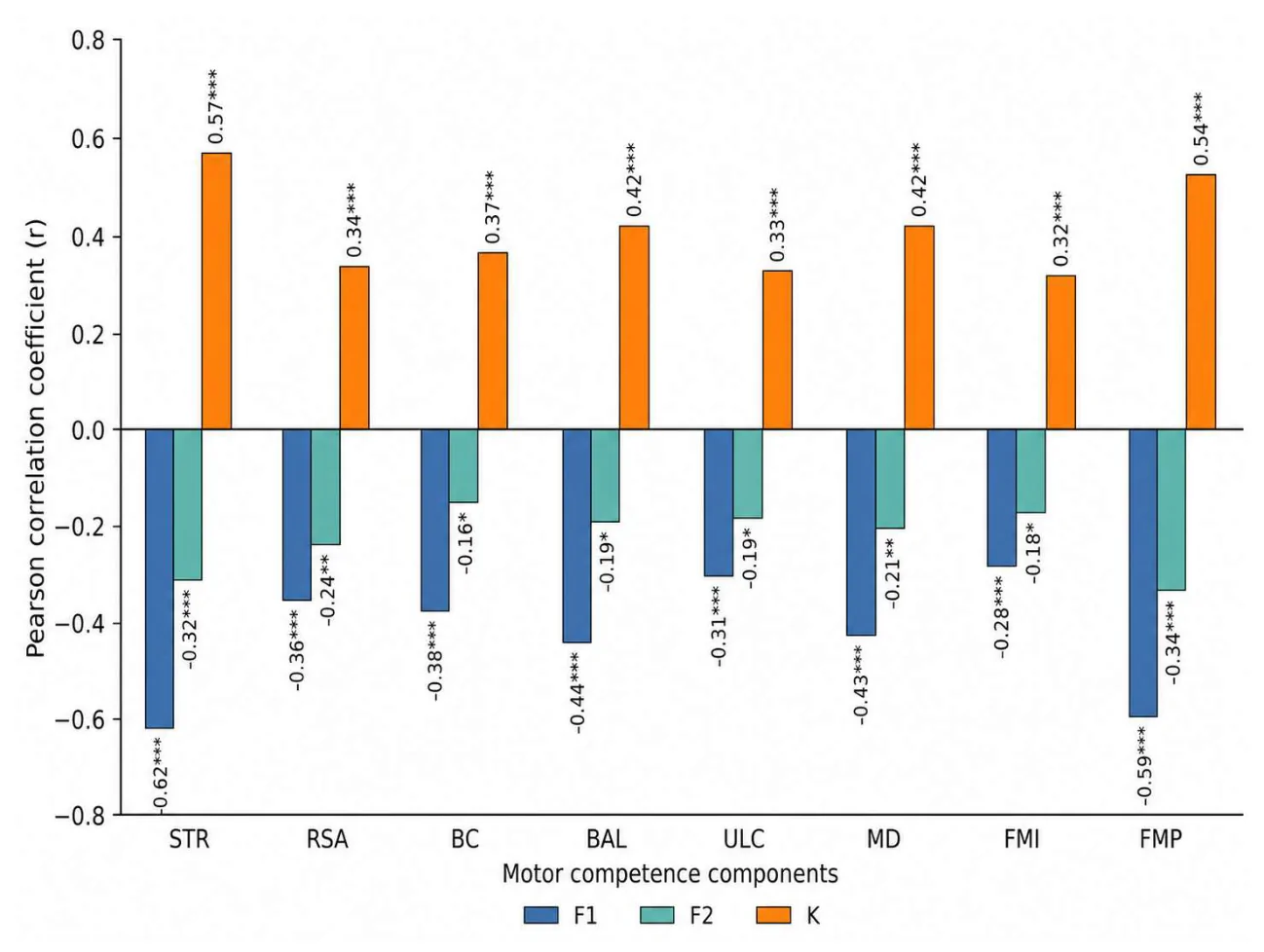
Correlations between BOT-2 motor competence components and phase-based motor learning outcomes. Note. F1 = time to initial acquisition; F2 = time to early refinement; K = final performance quality; FMP = Fine Motor Precision; FMI = Fine Motor Integration; MD = Manual Dexterity; ULC = Upper-Limb Coordination; BAL = Balance; BC = Bilateral Coordination; RSA = Running Speed and Agility; STR = Strength. * *p* < 0.05, ** *p* < 0.01, *** *p* < 0.001.

The correlation pattern is visually summarized in Figure 1.

Overall, correlations with F1 and K were more pronounced than correlations with F2.

### 3.3. Prediction of Phase-Based Motor Learning Outcomes

The hierarchical regression analyses across learning outcomes are summarized in Table 2. The models were adjusted for age and sex, and previous participation experience was included before motor competence components. Across all outcomes, participation experience showed the largest standardized coefficients among the retained predictors.

For Phase 1 (F1), participation experience showed the strongest negative association with learning time (β = −0.57, *p* < 0.001). Fine motor precision was also retained as an independent predictor (β = −0.24, *p* = 0.002), while bilateral coordination approached statistical significance (β = −0.14, *p* = 0.056). The final model explained 72.3% of the variance in F1, with an adjusted R^2^ of 0.703. The inclusion of participation experience produced the largest increase in explained variance (ΔR^2^ = 0.595), while the addition of BOT-2 components provided further explanatory value (ΔR^2^ = 0.111).

For Phase 2 (F2), participation experience showed the largest standardized coefficient (β = −0.45, *p* < 0.001). Fine motor precision (β = −0.23, *p* = 0.044) and running speed and agility (β = −0.19, *p* = 0.029) were also retained in the final model. The final model explained 33.7% of the variance in F2, with an adjusted R^2^ of 0.288. The inclusion of participation experience accounted for the largest increase in explained variance (ΔR^2^ = 0.276), whereas the addition of BOT-2 components provided a smaller incremental contribution (ΔR^2^ = 0.058).

For final performance quality (K), participation experience showed a positive association (β = 0.48, *p* < 0.001). No individual motor competence component reached conventional statistical significance when considered simultaneously with the other predictors, although fine motor precision (β = 0.17, *p* = 0.057) and bilateral coordination (β = 0.16, *p* = 0.066) approached significance. The final model explained 59.7% of the variance in K, with an adjusted R^2^ of 0.567. Participation experience again produced a substantial increase in explained variance (ΔR^2^ = 0.431), while the inclusion of individual BOT-2 components added further explanatory value (ΔR^2^ = 0.124).

### 3.4. Summary of Findings

In summary, previous participation experience was retained across all three hierarchical regression models. Among the BOT-2 motor competence components, fine motor precision was retained for F1 and F2 and showed a trend-level association with K; running speed and agility was retained for F2; and bilateral coordination showed trend-level associations with F1 and K.

## 4. Discussion

The present study examined the interrelationships among selected motor competence components and their phase-specific contribution to early motor learning outcomes in preschool children. Building on previous work showing that structured kindergarten kinesiology programs are associated with higher levels of motor skills [11] and that previous participation experience and baseline motor proficiency are related to phase-based motor skill acquisition [12], the present study focused on a more specific question: whether individual BOT-2-derived motor competence components contribute differently to initial acquisition, early refinement, and final performance quality.

The findings showed that motor competence components were positively interrelated, supporting the view that motor competence in preschool children is a structured and multidimensional construct rather than a set of isolated abilities [7,8,13]. The observed associations among fine motor precision, strength, balance, bilateral coordination, running speed and agility, and upper-limb coordination suggest that different domains of motor competence are functionally connected during early childhood. This is consistent with developmental perspectives emphasizing that motor competence develops through the interaction of multiple movement capacities, including balance, coordination, strength, locomotor control, and object-control abilities [1,4,5].

The correlation results further indicated that higher baseline motor competence was associated with shorter learning times for both Phase 1 and Phase 2 and with higher final performance quality. This finding is consistent with the assumption that children with stronger motor foundations are better prepared to acquire, organize, and refine new movement patterns [1,2,6]. It also supports previous findings from the same research line showing that higher BOT-2 motor proficiency is associated with more favorable phase-based motor learning outcomes in preschool children [12]. However, the present study extends this earlier evidence by showing that these associations are also visible at the level of specific motor competence components.

Participation experience showed the largest and most consistent associations across all learning outcomes. This result is in line with previous evidence showing that structured and repeated participation in organized physical activity programs is associated with more favorable motor competence outcomes in preschool children [3,9,10]. From a motor learning perspective, this finding is also consistent with the principle that repeated, structured, and appropriately organized practice supports the acquisition, stabilization, and refinement of motor skills [15,22]. It is further consistent with previous findings from this research line, where greater exposure to an organized kindergarten kinesiology program was associated with higher BOT-2 outcomes and more favorable phase-based motor learning indicators [11,12]. In the present study, participation experience accounted for the largest increase in explained variance across F1, F2, and K, suggesting that accumulated structured movement exposure is strongly related to children’s readiness to learn new motor tasks.

Although participation experience was the dominant predictor, selected BOT-2 components provided additional explanatory value. The inclusion of BOT-2 components in the final step of the hierarchical models increased the explained variance by 11.1% for F1, 5.8% for F2, and 12.4% for K. These values indicate that component-level motor competence adds information beyond age, sex, and previous participation experience, although the strength of this additional contribution differed across outcomes. This finding is relevant from a practical and curriculum-oriented perspective because it suggests that specific aspects of baseline motor competence may help explain individual differences in children’s readiness for new movement content. However, because of the observational design, these associations should not be interpreted as evidence that individual components directly cause faster learning or better final performance.

Fine motor precision was retained in the models for both initial acquisition and early refinement. This finding suggests that precision-related motor control may be relevant not only for fine motor tasks, but also for the broader organization and stabilization of new movement patterns. Initial acquisition requires the child to recognize the basic task structure, regulate movement direction, and produce a sufficiently accurate first successful execution [16,17,18,22]. Fine motor precision may therefore reflect a broader capacity for controlled, accurate, and goal-directed movement regulation that supports early learning across different task types.

The retention of fine motor precision in the Phase 2 model suggests that precision-related control may remain relevant after the first successful execution has been achieved. Early refinement requires the child to repeat the emerging movement pattern with greater stability, coordination, and control [15,16,17]. This interpretation is compatible with motor learning perspectives that emphasize feedback-based adjustment, error correction, and progressive stabilization of movement execution [15,22].

Running speed and agility was retained specifically in the model for Phase 2, but not for Phase 1 or final performance quality. This pattern may indicate that dynamic movement efficiency becomes more relevant during early refinement than during the first successful execution. Once the basic movement structure has been established, children may need greater speed, agility, coordination, and dynamic control to repeat and refine the movement pattern more efficiently.

In contrast, no single motor competence component reached conventional statistical significance as an independent predictor of final performance quality when considered simultaneously with participation experience and the other motor competence components. Although fine motor precision and bilateral coordination showed trend-level associations, these findings should be interpreted cautiously. This pattern may indicate that final performance quality reflects the integrated contribution of multiple motor domains rather than the isolated influence of a single component.

The hierarchical model fit statistics further support the phase-specific interpretation of the findings. The final models explained a substantial proportion of variance in F1 and K and a moderate proportion of variance in F2. Across all outcomes, participation experience accounted for the largest increase in explained variance, whereas individual BOT-2 components provided smaller but selective contributions. This pattern suggests that structured movement exposure provides a broad foundation for motor learning readiness, while specific motor competence components may contribute differently depending on the phase of learning. These findings are compatible with the challenge point framework, which emphasizes the importance of matching task demands to the learner’s skill level and readiness [19].

From a pedagogical perspective, the present findings have several implications for early childhood kinesiology programs. First, the results support the importance of sustained and structured movement experiences rather than short-term or fragmented exposure. Previous intervention literature has shown that structured physical activity programs can improve motor competence in children [3,10], while the present findings suggest that accumulated participation experience is also strongly related to the speed and quality of learning new motor tasks. This reinforces the need for continuous, planned, and developmentally progressive movement programs in kindergarten settings.

Second, the findings suggest that assessment-informed instruction may be more effective than programming based only on chronological age or general observation. Children of the same age may differ substantially in previous participation experience and in the structure of their motor competence profiles [11,12]. Therefore, practitioners should consider not only whether a child is generally motor competent, but also which specific components may support or limit learning in particular phases. For example, children with lower precision-related control may require additional support during initial acquisition and early refinement, whereas children with lower dynamic movement efficiency may require more targeted activities during refinement. This is particularly relevant because previous findings from the same program context suggested that additional years of participation may maintain acquired motor skill levels without necessarily producing proportional further improvements [11]. Therefore, phase-based assessment may help practitioners decide when continued consolidation is appropriate and when children may benefit from progression toward more complex movement tasks.

Third, the results support the need for program progression that includes diverse motor content and gradually increasing task demands. A multilateral approach that includes locomotor, manipulative, balance, coordination, strength, and object-control activities may provide a broad foundation for later motor learning [1,4,5]. However, the present findings suggest that this foundation should be complemented by structured assessment and individualized progression, so that task difficulty and instructional support are aligned with children’s actual motor readiness [19,20].

Such progression may also be relevant for children’s motivation during the learning process. If program content remains overly repetitive or insufficiently adapted to children’s current level of motor readiness, children may have fewer opportunities to experience optimal challenge, successful progression, and meaningful engagement with new movement tasks. In contrast, a systematically upgraded program that gradually introduces more complex motor tasks may help maintain children’s interest and support continued involvement in practice. This interpretation is consistent with the OPTIMAL theory of motor learning, which emphasizes that motivational and attentional factors, including enhanced expectancies, autonomy support, and externally directed attention, can facilitate motor performance and learning [23].

The present study contributes to the existing literature by shifting the focus from general motor proficiency and participation experience toward the internal structure of motor competence and its phase-specific relevance for learning. Previous work from this research line demonstrated that multiyear participation in organized kindergarten exercise programs is associated with motor skill outcomes [11], and that previous participation experience and total BOT-2 proficiency are related to phase-based motor skill acquisition [12]. The present study extends this line of evidence by showing that individual motor competence components do not contribute uniformly across learning outcomes, but instead show selective associations with different phases of motor learning.

Several limitations should be acknowledged. First, the observational analytical design does not allow causal conclusions. Although participation experience and motor competence components were associated with phase-based learning outcomes, longitudinal and experimental designs are needed to confirm the direction and mechanisms of these relationships. Second, the sample consisted of preschool children from a specific kindergarten and program context, which may limit generalizability to other educational settings, age groups, or populations with different levels of physical activity exposure. Third, the results derive from a specific 7-week motor learning protocol and from selected unfamiliar kinesiology tasks, meaning that the findings may not generalize to all forms of motor learning or to more sport-specific contexts.

Another limitation is that the phase-based outcomes depended on predefined task-specific criteria and expert video evaluation. Although inter-rater reliability was very high for the main phase indicators, future studies should continue to examine the sensitivity and reproducibility of this approach across different tasks and populations. In addition, several unmeasured factors may have influenced the observed associations. Biological maturation, cognitive development, attention during task practice, motivation, emotional readiness, previous informal movement experiences, and family or environmental context could affect both baseline motor competence and the speed or quality of learning new motor tasks. These factors were not included in the present regression models and should be considered in future longitudinal and experimental studies. Sex was included as a control variable, but sex-specific differences were not the primary focus of the present study. Future research should examine whether the phase-specific associations observed here differ by sex, age group, task type, or duration of structured movement exposure.

## 5. Conclusions

The present study showed that early motor learning in preschool children is associated not only with accumulated structured movement experience, but also with the internal structure of baseline motor competence. Previous participation experience in an organized kindergarten kinesiology program showed the largest and most consistent association with phase-based motor learning outcomes, suggesting that accumulated structured movement exposure is strongly related to the speed of initial acquisition, early refinement, and final performance quality.

Beyond participation experience, selected BOT-2 motor competence components showed phase-specific associations. Fine motor precision was independently associated with both Phase 1 and Phase 2, suggesting that precision-related motor control may be relevant for the establishment of the basic movement structure and for early refinement of execution. Running speed and agility were associated specifically with Phase 2, indicating that dynamic movement efficiency may become particularly important once the basic movement structure has been established. In contrast, no individual motor competence component independently predicted final performance quality at the conventional level of statistical significance. Final performance quality therefore appeared to be more closely related to accumulated structured movement experience and the integrated contribution of multiple motor domains than to any single isolated component.

These findings support the value of phase-based motor learning assessment in preschool settings. By identifying when children achieve initial acquisition and early refinement, practitioners may be better able to decide when to consolidate simple movement elements and when to progress toward more complex motor tasks. From a practical perspective, the results highlight the importance of developmentally progressive, structured, and assessment-informed kinesiology programs that consider both previous participation experience and specific motor competence profiles. Because the study design was observational, the findings should be interpreted as associations that can inform future intervention research rather than as evidence of direct causal effects.

## Figures and Tables

**Table 1:** Correlations between motor competence components and phase-based motor learning outcomes.

Outcome	STR	RSA	BC	BAL	ULC	MD	FMI	FMP
F1	−0.62 ***	−0.36 ***	−0.38 ***	−0.44 ***	−0.31 ***	−0.43 ***	−0.28 ***	−0.59 ***
F2	−0.32 ***	−0.24 **	−0.16 *	−0.19 *	−0.19 *	−0.21 **	−0.18 *	−0.34 ***
K	0.57 ***	0.34 ***	0.37 ***	0.42 ***	0.33 ***	0.42 ***	0.32 ***	0.54 ***

Note. F1 = time to initial acquisition; F2 = time to early refinement; K = final performance quality. FMP = Fine Motor Precision; FMI = Fine Motor Integration; MD = Manual Dexterity; ULC = Upper-Limb Coordination; BAL = Balance; BC = Bilateral Coordination; RSA = Running Speed and Agility; STR = Strength. * *p* < 0.05, ** *p* < 0.01, *** *p* < 0.001.

**Table 2:** Hierarchical regression models predicting phase-based motor learning outcomes.

Predictor	F1 β	F1 *p*	F2 β	F2 *p*	K β	K *p*
EXP	−0.57 ***	<0.001	−0.45 ***	<0.001	0.48 ***	<0.001
FMP	−0.24 **	0.002	−0.23 *	0.044	0.17	0.057
BC	−0.14	0.056	—	—	0.16	0.066
RSA	—	—	−0.19 *	0.029	—	—
Model R^2^	0.723		0.337		0.597	
Adjusted R^2^	0.703		0.288		0.567	
ΔR^2^ EXP	0.595		0.276		0.431	
ΔR^2^ BOT-2 components	0.111		0.058		0.124	

Note. β = standardized regression coefficient; F1 = time to initial acquisition; F2 = time to early refinement; K = final performance quality; EXP = participation experience; FMP = Fine Motor Precision; BC = Bilateral Coordination; RSA = Running Speed and Agility. Models were adjusted for age and sex. — indicates that the predictor was not retained in the final model. R^2^ = coefficient of determination; ΔR^2^ = change in explained variance after adding the respective model step. * *p* < 0.05, ** *p* < 0.01, *** *p* < 0.001.

## Data Availability

The data supporting the findings of this study are available from the author upon reasonable request.

## Supplementary Materials

The following supporting information can be downloaded at: https://www.mdpi.com/article/10.3390/jfmk11030303/s1, Table S1: Detailed Description of BOT-2 Subtests Used in the Study; Table S2: Standardized 7-Week Motor Learning Program Schedule; Table S3: Task-Specific Criteria for Assessing Phase-Based Motor Learning and Final Performance Quality.

## Abbreviations

The following abbreviations are used in this manuscript:

BOT-2	Bruininks–Oseretsky Test of Motor Proficiency, Second Edition
F1	Phase 1; time to initial acquisition
F2	Phase 2; time to early refinement
K	Final performance quality
FMP	Fine Motor Precision
FMI	Fine Motor Integration
MD	Manual Dexterity
ULC	Upper-Limb Coordination
BAL	Balance
BC	Bilateral Coordination
RSA	Running Speed and Agility
STR	Strength
EXP	Participation experience
