# Supplementary material for: Which Motor Competence Components Drive Early Motor Learning? A Phase-Based Analysis in Preschool Children
Source: J Funct Morphol Kinesiol. 2026 Jul 30;11(3):303. doi: 10.3390/jfmk11030303 (PMC13511555; doi:10.3390/jfmk11030303)
Supplement: Supplementary file 1 [file jfmk-11-00303-s001.zip › jfmk-4447568-supplementary.pdf]

Supplementary Materials

Supplementary Table S1 presents the selected BOT-2 subtests used to define the motor competence components analyzed in the present study.

Table S1. Detailed Description of BOT-2 Subtests Used in the Study.

Domain	Subtest (Code)	Description
Fine Motor Precision (FMP)	Drawing Lines Through Crooked Paths (MPCLZP)	The participant draws a line along a predefined curved path using the dominant hand, without crossing boundaries. Paper rotation >45° is not allowed.
Fine Motor Precision (FMP)	Folding Paper (MPPP)	The participant replicates demonstrated folds along marked lines on a sheet of paper.
Fine Motor Integration (FMI)	Copying a Square (MIPK)	The participant reproduces a square using the dominant hand without rotating the paper beyond 45°.
Fine Motor Integration (FMI)	Copying a Star (MIPZ)	The participant reproduces a star shape under the same conditions.
Manual Dexterity (MD)	Transferring Pennies (AMSN)	The participant transfers coins from the dominant to the non-dominant hand into a container within 15 s.
Upper-Limb Coordination (ULC)	Dropping and Catching a Ball (KRPHL)	The participant drops and catches a ball with both hands after a bounce.
Upper-Limb Coordination (ULC)	Dribbling with Alternating Hands (KRNVL)	The participant performs controlled dribbling while alternating hands.
Balance (BAL)	Walking Forward on a Line (RVHL)	The participant walks along a straight line, placing each foot fully on the line.
Balance (BAL)	Standing on One Leg (RVJSG)	The participant maintains balance on a beam on one leg with eyes open.
Bilateral Coordination (BC)	Jumping in Place (BKSPM)	The participant performs coordinated jumps with alternating arm and leg positions.
Bilateral Coordination (BC)	Synchronized Tapping (BKSTNR)	The participant performs synchronized tapping of hands and feet on the same body side.
Running Speed and Agility (RSA)	One-Legged Hop (BAPJN)	The participant performs as many hops as possible on one leg within 15 s.
Strength (STR)	Knee Push-Ups (SNSK)	The participant performs push-ups on knees within 30 s.
Strength (STR)	Sit-Ups (SNPT)	The participant performs sit-ups within 30 s.

Supplementary Table S2 presents the implementation schedule of the standardized 7-week motor learning program applied to all participants after baseline BOT-2 assessment. The program consisted of 14 sessions and included nine unfamiliar motor tasks of different structural complexity. Each session combined newly introduced tasks with play-based activities. Sessions marked with "Final Performance Assessment (K)" indicate the session in which the final performance quality of the relevant task was evaluated.

Table S2. Standardized 7-Week Motor Learning Program Schedule.

(A) Session-by-Session Schedule of the Experimental Program

Session	New Motor Tasks	Additional Task	Play Activity
1	1. Jumping rope in place; 8. Shoulder blade stand	-	Game 18
2	1. Jumping rope in place; 8. Shoulder blade stand	Final Performance Assessment (K) for Task 1	Game 19
3	2. Jumping rope while moving forward; 8. Shoulder blade stand	-	Game 18
4	2. Jumping rope while moving forward; 8. Shoulder blade stand	Final Performance Assessment (K) for Task 2	Game 19
5	3. Ball toss and catch in place; 8. Shoulder blade stand	-	Game 19
6	3. Ball toss and catch in place; 8. Shoulder blade stand	Final Performance Assessment (K) for Tasks 3 and 8	Game 21
7	4. Ball toss and catch while moving; 9. Sambon Tsuki	-	Game 21
8	4. Ball toss and catch while moving; 9. Sambon Tsuki	Final Performance Assessment (K) for Task 4	Game 22
9	5. Two-foot vault over a gym bench from arm support; 9. Sambon Tsuki	-	Game 20
10	5. Two-foot vault over a gym bench from arm support; 9. Sambon Tsuki	Final Performance Assessment (K) for Task 5	Game 17
11	6. Carrying a 1 kg bag on the head; 9. Sambon Tsuki	-	Game 18
12	6. Carrying a 1 kg bag on the head; 9. Sambon Tsuki	Final Performance Assessment (K) for Task 6	Game 20
13	7. Crawling through hoops feet-first from front-arm support; 9. Sambon Tsuki	-	Game 17
14	7. Crawling through hoops feet-first from front-arm support; 9. Sambon Tsuki	Final Performance Assessment (K) for Tasks 7 and 9	Game 21

(B) Program Design Notes

- All participants completed the same program under identical instructional conditions, regardless of their previous experience of participation in the kindergarten kinesiology program.
- The nine motor tasks were intentionally selected as unfamiliar to the children and had not been practiced previously within either the regular preschool educational program or the structured kindergarten kinesiology program.
- The program was designed to combine less demanding tasks (simple tasks) with more demanding tasks (complex and more complex tasks) across the weekly schedule, in order to maintain developmental appropriateness and observation continuity.
- Task performance was video recorded during the program, and delayed expert analysis was used to determine Phase 1 (F1), Phase 2 (F2), and final performance quality (K).

Legend: Tasks 1-7 represent simple motor tasks, Task 8 represents the complex motor task, and Task 9 represents the more complex motor task. K refers to the final performance quality assessment conducted at the end of the final scheduled exposure for the relevant task.

Supplementary Table S3 presents the operational criteria used to assess phase-based motor learning in the nine novel motor tasks included in the study. For each task, a brief task description is provided together with the predefined criteria for Phase 1 (F1; initial structural acquisition) and Phase 2 (F2; initial refinement). Final performance quality (K) was evaluated using a standardized 5-point expert rating scale applied across all tasks.

Table S3. Task-Specific Criteria for Assessing Phase-Based Motor Learning and Final Performance Quality.

(A) General Performance Quality Criteria Applied Across All Tasks

Score	General Criterion for Final Performance Quality (K)
5	The child performs the task correctly, fluently, and confidently, without relevant technical or aesthetic errors. Movement is coordinated, stable, and clearly recognizable according to the intended task structure.
4	The child performs the task mostly correctly, with minor technical or aesthetic deviations. Movement is generally coordinated and recognizable, with only slight instability, timing delay, or reduced precision.
3	The child performs the task with noticeable technical or aesthetic errors. The basic movement pattern is recognizable, but coordination, fluency, timing, or control is inconsistent.
2	The child performs the task in a markedly crude, unstable, or incomplete manner that substantially deviates from the intended movement structure. The movement can be initiated or partially completed, but execution is fragmented, hesitant, or repeatedly interrupted.
1	The child is unable to perform the task, even with assistance, or the task structure is not recognizably achieved.

(B) Task-Specific Criteria for Phase 1 and Phase 2

No.	Motor Task	Brief Task Description	F1 Criterion	F2 Criterion	Task-Specific Notes for Quality
1	Jumping Rope in Place	The child repeatedly jumps over a short rope in place using a two-foot take-off, with synchronized arm action.	First successful two-foot jump over the rope.	Coordinated execution of multiple consecutive rope jumps with synchronized arm movement.	Emphasis on arm-leg coordination, rhythm continuity, and absence of repeated rope stoppage.
2	Jumping Rope While Moving Forward	The child repeatedly jumps over a short rope while progressing forward.	First successful rope jump while moving forward.	Coordinated execution of multiple consecutive rope jumps while moving forward with synchronized arm action.	Emphasis on continuity of forward progression and preserved jumping rhythm.
3	Ball Toss and Catch in Place	The child repeatedly tosses a ball above the head and catches it with both hands while standing in place.	First successful toss and catch with both hands.	Coordinated execution of six or more consecutive toss-and-catch repetitions.	Emphasis on bilateral coordination, timing control, and rhythmic continuity.
4	Ball Toss and Catch While Moving	The child repeatedly tosses a ball above the head and catches it with both hands while moving forward.	First successful toss and catch with both hands while moving.	Coordinated execution of six or more consecutive toss-and-catch repetitions while maintaining forward movement.	Emphasis on simultaneous locomotor and object-control coordination.
5	Two-Foot Vault Over Bench from Arm Support	The child transfers both feet from one side of a bench to the other while maintaining arm support.	First successful two-foot transfer while maintaining arm support.	Coordinated execution of five or more consecutive transfers.	Emphasis on stable arm support, coordinated leg transfer, and movement continuity.
6	Carrying a 1 kg Bag on the Head	The child walks forward while carrying a 1 kg bag on the head without dropping it.	First successful completion of two marked path lengths without stopping and without dropping the bag.	Successful completion of six marked path lengths without stopping and without dropping the bag.	Emphasis on postural control, head stability, and continuity of walking.
7	Crawling Through a Hoop Feet-First from Front-Arm Support	From a front-arm support position, the child moves forward and passes through a hoop feet-first without the hips touching the floor.	First successful passage through the hoop without hip contact with the floor.	Successful completion of four or more repetitions without hip contact with the floor.	Emphasis on trunk control, support stability, and smooth forward movement.
8	Shoulder Blade Stand	From a seated position, the child lifts the legs upward into a shoulder blade stand and supports the hips with the hands.	First successful holding of the correct position for >3 s; legs partially extended, but with limited stability.	Successful holding of the correct position for >6 s; legs mostly extended, with visibly better stability and control.	Emphasis on alignment, hold duration, trunk stability, and control.
9	Sambon Tsuki	The child moves forward step by step and performs three consecutive arm strikes after each step.	First successful execution in the correct position, with an appropriate step and synchronized arm action.	Successful execution of four consecutive repetitions in the correct position, with stable stepping and synchronized arm action without visible stopping.	Emphasis on arm-leg synchronization, rhythmic sequencing, trunk control, and balance.

Note: The same general expert-based framework was applied across all tasks. Task-specific execution features were interpreted within this shared rating structure. Legend: Phase 1 (F1) refers to the first observable successful execution of the essential movement structure according to predefined task-specific criteria. Phase 2 (F2) refers to the first observable point at which the child demonstrated a more stable, coordinated, and repeatable execution pattern according to predefined task-specific criteria. K refers to final performance quality, rated on a 5-point expert scale.
